# Genetic factors explain a significant part of associations between adolescent well-being and the social environment

**DOI:** 10.1007/s00787-021-01798-3

**Published:** 2021-05-24

**Authors:** Margot P. van de Weijer, Dirk H. M. Pelt, Catharina E. M. van Beijsterveldt, Gonneke Willemsen, Meike Bartels

**Affiliations:** 1 Department of Biological Psychology, Vrije Universiteit Amsterdam, van der Boechorststraat 7, 1081 BT Amsterdam, The Netherlands; 2 Amsterdam Public Health Research Institute, Amsterdam University Medical Center, Amsterdam, The Netherlands

**Keywords:** Well-being, Adolescence, Family environment, Friendships, Heritability

## Abstract

Socio-environmental factors play an important role in adolescent well-being, but potential genetic contributions to these associations are rarely assessed. To address this gap in the literature, associations between well-being and family conflict and functioning, number of friends, friendship importance and satisfaction, and leisure time variables were studied in *N* =  ~ 4700 twin pairs from the Netherlands Twin Register, us ing generalized estimating equations and twin-difference scores. When twin-difference scores indicated a role for genetic factors, we used bivariate genetic models to quantify genetic and environmental contributions to these associations. We identify significant associations between well-being and family functioning, family conflict, different leisure time activities, number of friends, and satisfaction with friendships. Additionally, we find evidence for large (73–91%) genetic influence on the associations between well-being and family conflict and functioning, leisure time sport/scouting clubs, and satisfaction with friendships. Finally, findings support the hypothesis of a causal association between well-being and family conflict and functioning. These findings have important implications for research into the social correlates of well-being in adolescence, as not taking genetic factors into account leads to overestimations of the influence of identified correlates and consequently to recommendations of these correlates as intervention targets.

**Supplementary Information:**

The online version contains supplementary material available at 10.1007/s00787-021-01798-3.

## Introduction

Adolescence, defined by the WHO as the period between age 10 and 19, marks a period in life where a person transitions from childhood to adulthood. During this transition period, the body rapidly develops, and there is accumulating evidence that adolescence is a critical period for later health and disease [1]. For example, half of the cases of lifetime DSM-IV anxiety, mood, impulse control, and substance use disorders have had their onset by age 14. While this period of pubertal mental and bodily maturation thus represents a period full of risk, it can also be interpreted as a period that holds great potential for interventions.

The focus of adolescent mental health research so far has mainly been on mental illness. For example, there is abundant research into how depression in adolescence might lead to adult depression, comorbid disorders, and suicide [2–5]. With this emphasis on mental illness, it is easily forgotten that most adolescents develop relatively well, with only a small proportion of adolescents reporting low levels of well-being [6, 7]. In addition, large genetically informed studies find genetic correlations of ~ 0.7 between well-being and depression, suggesting that, although they are substantially related, the genetic predisposition for well-being is partly independent from the genetic predisposition for depression [8, 9] and that well-being is more than just the absence of depression. Therefore, in addition to studying mental illness and its risk factors, it is valuable to study the determinants of mental health and well-being.

Creating adolescent interventions to improve adult outcomes requires in-depth understanding of the determinants of adolescent well-being. This is supported by findings that adolescent well-being predicts adult well-being and general health [10, 11]. Given the importance of adolescent well-being for later-in-life outcomes, it is essential to identify its correlates and determinants. One of the most studied factors in relation to well-being is one’s social environment. For example, a meta-analysis on the associations between well-being and social support measures in children and adolescents across 246 studies found that social support from parents, peers, and teachers is positively associated with well-being [12]. Moreover, a review focusing on the connection between well-being and friendships concludes that children’s friendships are associated with their happiness, and that negative social relationships have an adverse effect on their well-being [13].

The nature of these associations, however, often remains unexplored. Are these relations causal or is there an unmeasured third factor that is related to both, resulting in the observed association? For example, resilience and well-being are often observed to be strongly associated, accompanied by firm conclusions about the direction of causation. However, 51% of the phenotypic association between resilience and well-being is accounted for by a third factor: genetic influences [14]. For socio-environmental factors, it was traditionally assumed that epidemiological associations between individuals and their environment could only be explained in a unidirectional manner, with the environment affecting the individual [15]. We have since learned that these associations are bidirectional, with environments also being subject to heritable influences, through individuals’ behavior [15–17]. Research showed that the heritability of well-being is 40% [18, 19], meaning that about 40% of the individual differences in well-being can be explained by genetic differences between people. Thus, if we study well-being in relation to another heritable trait (e.g., family conflict [16]), it may occur that the observed phenotypic association is (partly) due to overlapping genetic factors. If these genetic factors are not taken into account, one might overestimate the (causal) influence of identified social factors and consequently recommend these correlates as targets for interventions, even though they carry small or no direct (causal) effect on well-being.

In the current study, we investigate the underlying sources of associations between adolescent well-being and various socio-environmental factors. Using twin data from the Netherlands Twin Register (NTR), we examine monozygotic (MZ) and dizygotic (DZ) difference scores to explore the possibility that these associations are (partly) attributable to genetic factors. When evidence for genetic influences is seen, we use bivariate genetic models to quantify the genetic and environmental influences on the covariance between well-being and socio-environmental factors.

## Materials and methods

### Sample

Study participants are voluntarily registrants at the NTR [20]. We selected a subset of NTR participants who filled out the Dutch Health and Behavior Questionnaire, administered to adolescent participants aged 13–17 (for more data collection information, see [21]). In total, well-being data were available for 11,406 adolescents from 4739 complete twin pairs and 1928 incomplete twin pairs (*M* age = 15.66, *SD* age = 1.31, *N* males/females = 4855/6551). Sample size per analysis varied depending on sample size available per social variable (see Online Resources, eTable 1). For each social variable, the sample size reflects the number of complete twin pairs that also have well-being data. If an individual had data available at more than one time-point, we used data from the last time-point. We made sure that within twin pairs, data were selected from the same time-point to reduce bias due to differences in timing of the survey. Zygosity in same-sex twin pairs was determined based on DNA genotyping (34.4%) or, when DNA samples were not available, by previously collected questionnaires containing parental-reports about same-sex twin similarity in physical characteristics and frequency of mistaking one twin for another by parents, relatives, and strangers. Based on these self-report questions, the accuracy of classification is 95.9% [20].

### Variables

*Well-being* was assessed using the Dutch version of the satisfaction with life (SWL) scale [22]. This scale contains five items that assess SWL on a 7-point Likert scale. The scale has good internal consistency in the sample (*α* = 0.87). Scores on the individual items are summed to create SWL scores for each respondent. An example of an item is: ‘I am satisfied with my life’. Well-being scores were standardized to z-scores in all analyses.

In the Dutch Health and Behavior Questionnaire (DHBQ)*, social variables* are available for the following categories: leisure time activities, family functioning, family conflict, and friendships. Scores for all variables were standardized to z-scores in all analyses.

*Leisure time activities* (LT) are assessed by self-report on how much time participants spend on the following activities: a) watching TV–videos–DVDs, b) computer games, c) computer/Internet d) making music/choir, e) reading, f) drawing/painting, g) handicrafts, h) being at home with friends, i) visiting friends, j) on the street with friends, k) sports club or scouting, l) chess, board games, and m) going out (disco, cafe, bar). For each activity, participants can choose from the following answer categories: 1) never, 2) only once until now, 3) less than once a week, 4) once a week, 5) a few days per week, 6) almost every day, and 7) every day. Since some activities can be categorized under broader categories, we summed some of the categories together into 1) computer games and computer/the Internet, 2) reading and chess, board games (hereafter referred to as indoor games), 3) drawing/painting and handicrafts, and 4) being at home with friends, visiting friends, and on the street with friends.

*Family functioning* is assessed using a Dutch translation of the subscale General Functioning of the Family Assessment Device (FAD) [23]. This 12-item scale measures overall (un)healthy family functioning, with items assessing problem solving, communication, roles, affective responsiveness, affective involvement, and behavior control. The subscale holds high reliability in our sample (*α* = 0.88). The items are answered on a scale from 1 to 4, 1 representing strong agreement with the item, and 4 representing strong disagreement. Since 6 items measure healthy functioning, and 6 measure unhealthy functioning, we recoded half of the items as 5—[item score], so that all questions were scored in the same direction. After this transformation, items are summed to create a total family functioning score, with higher scores indicating higher levels of dysfunction. An example of an item is ‘planning family activities is difficult, because we misunderstand each other’.

*Family conflict* is assessed using a Dutch translation of the subscale Conflict of the Family Environment Scale (FES) [24]. The subscale contains 11 items with a 2-point scale, with 1 = No and 2 = Yes. For each item, the participant indicates whether the presented statement is true for their family. The subscale shows acceptable reliability in our sample (*α* = 0.0.73). An example of item is: “In our family we argue a lot”. One item (“We seldom get openly angry at each other at home”) is reverse-coded, so that answering yes implies low family conflict, whereas yes on the other items indicates high family conflict. The Dutch translation of this item was misinterpreted by a lot of participants, leading to inconsistent data patterns or missing data [25]. Therefore, during data collection, this item was changed to “we often get openly angry at each other at home”, so that all items were collected in the same direction. For this study, we used this reworded version of the item. Scores on the 11 items were summed to get a total score for family conflict, with higher scores indicating higher levels of conflict.

*Friendship* is assessed in three ways: 1) “How many good male/female friends do you have?”; 2) “In general, how satisfied are you with your female/male friends?”, and 3) “In general, how important are your female/male friends to you?”. To minimize the number of statistical tests, we summed the responses for male and female friends for (1), and took the mean for (2) and (3). For question 1, the participant could answer within the following categories: 0) I do not have any good friends; 1) 1 or 2; 2) 3 or 4; 3) 5 or 6; 4) 7 or 8; 5) 9 or 10; 6) 11 to 15; 7) more than 15. For questions (2) and (3), the answering categories were as follows: 0) very dissatisfied/unimportant; 1) dissatisfied/unimportant, 2) somewhat satisfied/ important; 3) satisfied/important; 5) very satisfied/important.

### Statistical analyses

#### Phenotypic associations

Using the full sample (including incomplete twin pairs, see Table 1), we applied linear regression analysis to identify associations between well-being and the social variables. To correct for familial dependency in the observations, we used the generalized estimating equation (GEE) function in R [26]. In GEE, an exchangeable conditional covariance matrix is used to account for relatedness, and tests are based on sandwich-corrected, robust standard errors [27]. Sex was included as a covariate in the regression analyses.

**Table 1:** Associations between well-being and all variables

	GEE (whole sample)			DZ difference			MZ difference		
	*β* (SE)	*p*	*N*	*β* (SE)	*p*	*N* (pairs)	*β* (SE)	*p*	*N* (pairs)
FAD (family functioning)	− 0.35 (.01)	2.23 × 10^–181*^	10,478	− 0.23 (.02)	< 2 × 10^–16*^	2493	− 0.15 (.02)	3.18 × 10^–10*^	1596
FES (family environment)	− 0.26 (.01)	2.25 × 10^–98*^	7479	− 0.20 (.02)	< 2 × 10^–16*^	1707	− 0.13 (.03)	2.14 × 10^–6*^	1088
Leisure time—indoor games	0.03 (.01)	7.43 × 10^–4*^	11,044	0.02 (.02)	0.341	2707	− 0.01 (.02)	0.758	1768
leisure time—contact with friends	0.05 (.01)	4.60 × 10^–6*^	10,965	0.05 (.02)	.008	2689	0.003 (.02)	0.886	1716
Leisure time—crafts	− 0.01 (.01)	0.145	11,100	− 0.01 (.02)	0.457	2744	0.01 (.02)	0.622	1775
Leisure time—making music/choir	.004 (.01)	0.715	11,178	− 0.06 (.02)	.004	2769	0.01 (.02)	0.558	1798
Leisure time—computer	− 0.02 (.02)	0.278	3553	0.04 (.03)	0.285	1006	0.02 (.04)	0.559	679
Leisure time—going out (dancing)	0.03 (.01)	0.005	11,197	0.01 (.02)	0.467	2784	0.01 (.02)	0.735	1800
Leisure time—sport/scouting club	0.13 (.01)	3.70 × 10^–33*^	11,182	0.08 (.02)	8.79 × 10^–5*^	2776	0.03 (.02)	0.224	1806
Leisure time—TV	0.03 (.02)	0.072	3608	0.03 (.03)	0.391	1041	− 0.06 (.04)	0.155	701
Number of friends	0.12 (.01)	1.25 × 10^–21*^	7690	0.06 (.02)	.005	1771	0.07 (.03)	0.012	1143
Importance of friendships	0.06 (.03)	0.062	1322	− 0.05 (.06)	0.406	293	0.09 (.06)	0.143	209
Satisfaction with friendships	0.16 (.01)	9.33 × 10^–27*^	6519	0.12 (.03)	6.65 × 10^–6*^	1331	0.02 (.03)	0.468	863

*GEE* generalized estimating equation, *DZ* dizygotic, *MZ* monozygotic, *β* beta, *SE* standard error, *p* *p *value, *N* sample size

*Significant after correction for multiple testing (*α* = .0038)

#### Intra-pair difference scores

**Fig. 1:**
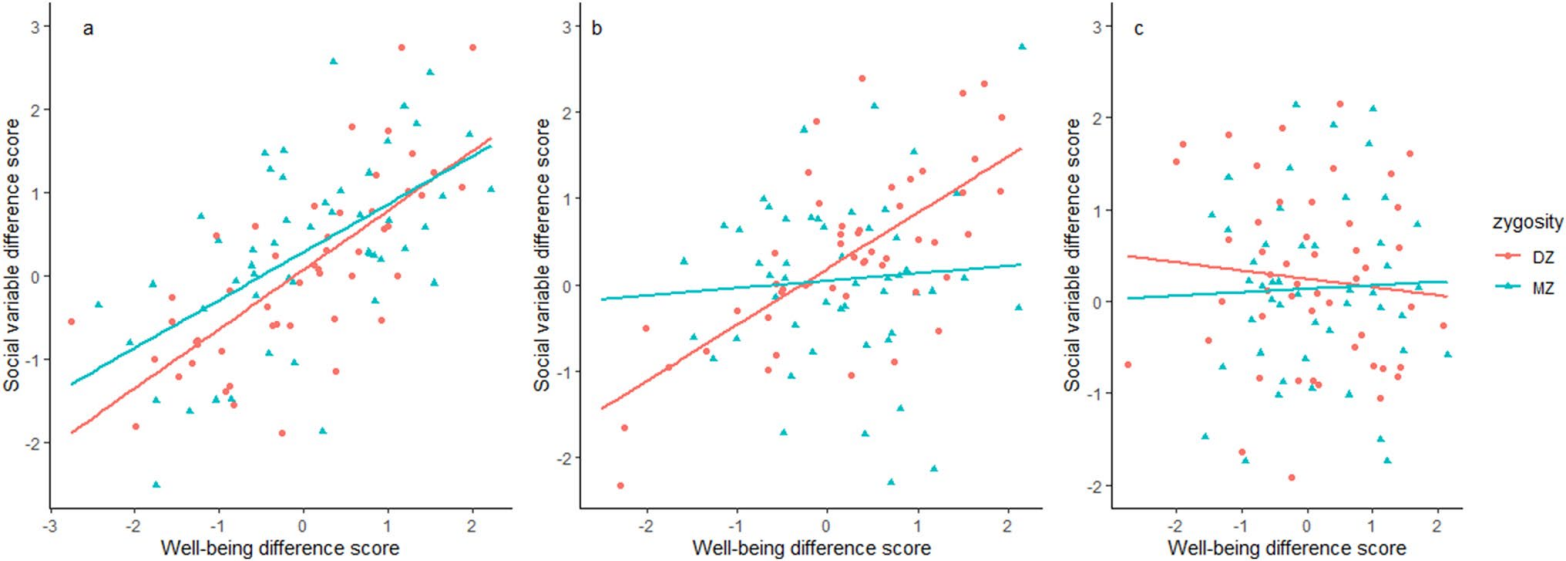
Expectations based on different scenarios: **a** significant MZ and DZ difference score associations suggest a causal effect between well-being and a social variable; **b** a significant DZ difference score association but no MZ difference score association suggests a large role for genetic factors; **c** lack of association of both MZ and DZ difference scores suggests a large role for common environmental influences

Intra-pair difference scores were used to get a first indication of the nature of the association between well-being and different social variables. Since MZ twins share both their genetic makeup (additive genetic effects A) and their common environment (C), intra-pair difference scores between the twins must be the result of unique environmental experiences (E). On the other hand, intra-pair difference scores in DZ twins can be a result of differences in unique environmental influences (E), but also a result of differences in their genetic makeup (A), since they only share 50% of their genetic material on average. In a twin-difference design, intra-pair difference scores for one trait are regressed on the intra-pair difference scores of another trait. Based on these analyses, we expect the following given there is an observed phenotypic association between the traits (see Fig. 1):

I. If there is a significant association between the intra-pair difference score of well-being and the intra-pair difference score of a social variable in both MZ and DZ twins, this supports the hypothesis there is a causal relation between the two traits, or a large role for E. Genetic factors are additionally likely to contribute when the absolute DZ regression coefficient is larger than the absolute MZ regression coefficient.

II. If there is a significant association between the intra-pair difference score of well-being and the intra-pair difference score of a social variable in DZ twins solely, it suggests that A plays a large role in the association, supporting the hypothesis that genetic factors act as a third unobserved variable underlying the association.

III. If in both MZ and DZ twins, the intra-pair difference score regression returns non-significant results, the covariance between two traits is likely caused by C as these are 100% shared in both types of twin pairs.

We calculated difference scores for all variables in all twin pairs by subtracting the score of one twin from the score of the other twin. Next, for each social variable, we regressed the social variable difference score on the well-being difference score using linear regression while correcting for a variable that reflects whether the twin pair is same sex or different sex. Since MZ twins are always same sex, we did not control for sex in MZ difference score analyses. We used a significance threshold of *α* = 0.05/13 = 0.0038 to correct for multiple testing (13 tests in total).

#### Bivariate twin models

For associations where we find significant intra-pair difference regression results in both MZ and DZ twins or in DZ twin solely, we use bivariate genetic models to quantify genetic and environmental influences on the covariance between well-being and social variable. The difference in genetic relatedness between MZ and DZ twins enables decomposing the (co)variance of the traits under investigation into additive genetic (A), dominant genetic (D), common environmental (C), and unique environmental factors (E, including measurement error). Since C and D cannot simultaneously be estimated based on MZ and DZ covariance alone, either an ACE or ADE model is fit. When the MZ correlation is more than twice the DZ correlation, an ADE model is fit. When the MZ correlation is less than twice the DZ correlation, an ACE model is fit. Based on the literature, it was likely that the twin correlations for well-being might suggest an influence of D [28], while the twin correlations for some social traits might suggest an influence of C [25]. As we cannot model both C and D in the bivariate model, and since we are most interested in potential common environmental influences, we a priori chose to model bivariate ACE models (see Online Resources, eFigure 1).

Twin correlations and cross-twin–cross trait (CT–CT) correlations were estimated in saturated models in which all parameters (means, variances, and covariances) are freely estimated. We modeled variance components separately for males and females. For satisfaction with friendships, scores were highly skewed. To prevent bias [29], we transformed this variable into an ordinal variable with three categories (low, middle, and high), and applied a liability threshold model with 2 thresholds. Under this model, it is assumed that there is an underlying continuous liability distribution for this trait, with two thresholds that that define three categories. The thresholds divided the data into three groups of equal sizes (33%).

Since the variance component approach does not yet allow for the inclusion of opposite-sex twins in estimating the variance components, DZ opposite-sex twins were excluded when estimating the variance components. We therefore could not test for qualitative sex differences. To test for quantitative sex differences (i.e., the same genetic and environmental factors exert influence of different magnitudes in males and females), we constrained variance components to be equal across males and females and compared the fit of the constrained model to that of the less restrictive model. Next, we tested whether C significantly contributed to the (co)variance by dropping common environmental components in three steps (see Online Resources, eFigure 1): 1) we dropped C for well-being (*c22*), 2) we dropped the covariance explained by C (*c21*), and 3) we dropped C for the social variable (*c11)*. If a C component could not be dropped for both sexes, we tested whether it could be dropped for males or females only. Additionally, we computed genetic and environmental correlations, reflecting the extent to which there is overlap in the latent genetic and environmental factors influencing the traits. Parameters were estimated using maximum-likelihood estimation in OpenMx [30] using the variance component approach [31]. By fitting the model with and without the constraints of interest, a log-likelihood ratio test can be used to compare models. The more parsimonious model is rejected if the log-likelihood statistic exceeds the chosen threshold. In line with the reasoning by Benjamin and colleagues [32] that the traditional p value threshold of 0.05 leads to a high false-positive rate, we used a *p* value threshold of *α* = 0.005.

## Results

### Phenotypic associations

In the GEE analyses (Table 1), higher well-being was significantly associated with less family dysfunction (FAD, *β* = -0.35, SE = 0.01, *p* = 2.23 × 10^–181^), less family conflict (FES, *β* = − 0.26, SE = 0.01, *p* = 2.25 × 10^–98^), more leisure time indoor games (*β* = 0.03, *SE* = 0.01, *p* = 0.7.43 × 10^–4^), more leisure time contact with friends (*β* = *0.0*5, SE = 0.01, *p* = 4.60 × 10^–6^), more leisure time sports club/scouting (*β* = 0.13, SE = 0.01, *p* = 3.70 × 10^–33^), a higher number of friends (*β* = 0.12, SE = 0.01, *p* = 1.25 × 10^–21^), and higher satisfaction with friendships (SWF) (*β* = 0.16, SE = 0.01, *p* = 9.33 × 10^–27^). Leisure time crafts, leisure time making music, leisure time computer, leisure time going out, leisure time TV, and importance of friendships were not associated with well-being.

### Intra-pair difference scores

Differences scores for two social variables were significantly associated with well-being difference scores in both MZ and DZ twin pairs: the FAD difference score (MZ: *β* = − 0.15, *p* = 3.18 × 10^–10^, DZ: *β* = − 0.23*, p* =  < 2 × 10^–16^) and the FES difference score (MZ: *β* = − 0.13, *p* = 2.14 × 10^–6^, DZ: *β* = − 0.20, *p* =  < 2 × 10^–16^). This supports the hypothesis that there is either a causal relation between the two traits, or a large role for E. Given that the DZ difference scores are more strongly associated than the MZ difference scores, there is also a potential role for A.

Difference scores for two variables were significantly associated with well-being difference scores in DZ twins, but not MZ twins: leisure time sport/scouting club (*β* = 0.08, *p* = 8.79 × 10^–5^), and the SWF difference score (*β* = 0.12, *p* = 6.65 × 10^–6^). This suggests that A plays a large role in the association between well-being and these social variables.

Finally, three of the variables for which we observed a phenotypic association with well-being were not significant in the intra-pair difference score analyses for both MZ and DZ twins (Table 1): leisure indoor games (MZ *β* = -0.01, *p* = 0.758, DZ: *β* = 0.02, *p* = 0.341), leisure time contact with friends (MZ: *β* = 0.003, *p* = 0.886, DZ: *β* = *0.0*5, *p* = 0.008), and number of friends (MZ: *β* = 0.07, *p* = 0.012, DZ: *β* = 0.06, *p* = 0.005). The lack of MZ and DZ difference score association indicates that shared environmental factors are likely the underlying source of the observed association between well-being and these social variables.

### Bivariate twin models

Based on the difference score analyses, four traits were followed up with bivariate genetic model fitting: FAD, FES, leisure time spend at sports/scouting club, and satisfaction with friendships. From the saturated models (model fitting results in Online Resources, eTable 2), we estimated the cross-twin and CT–CT correlations for each trait (Online Resources, eTable 3). For all traits, MZ correlations were higher than DZ correlations, indicating a role for A. Twin correlations for well-being and satisfaction with friendships in males indicated a potential role for D in the variance decomposition. However, as explained in the methods section, we only fit bivariate ACE models.

**Fig. 2:**
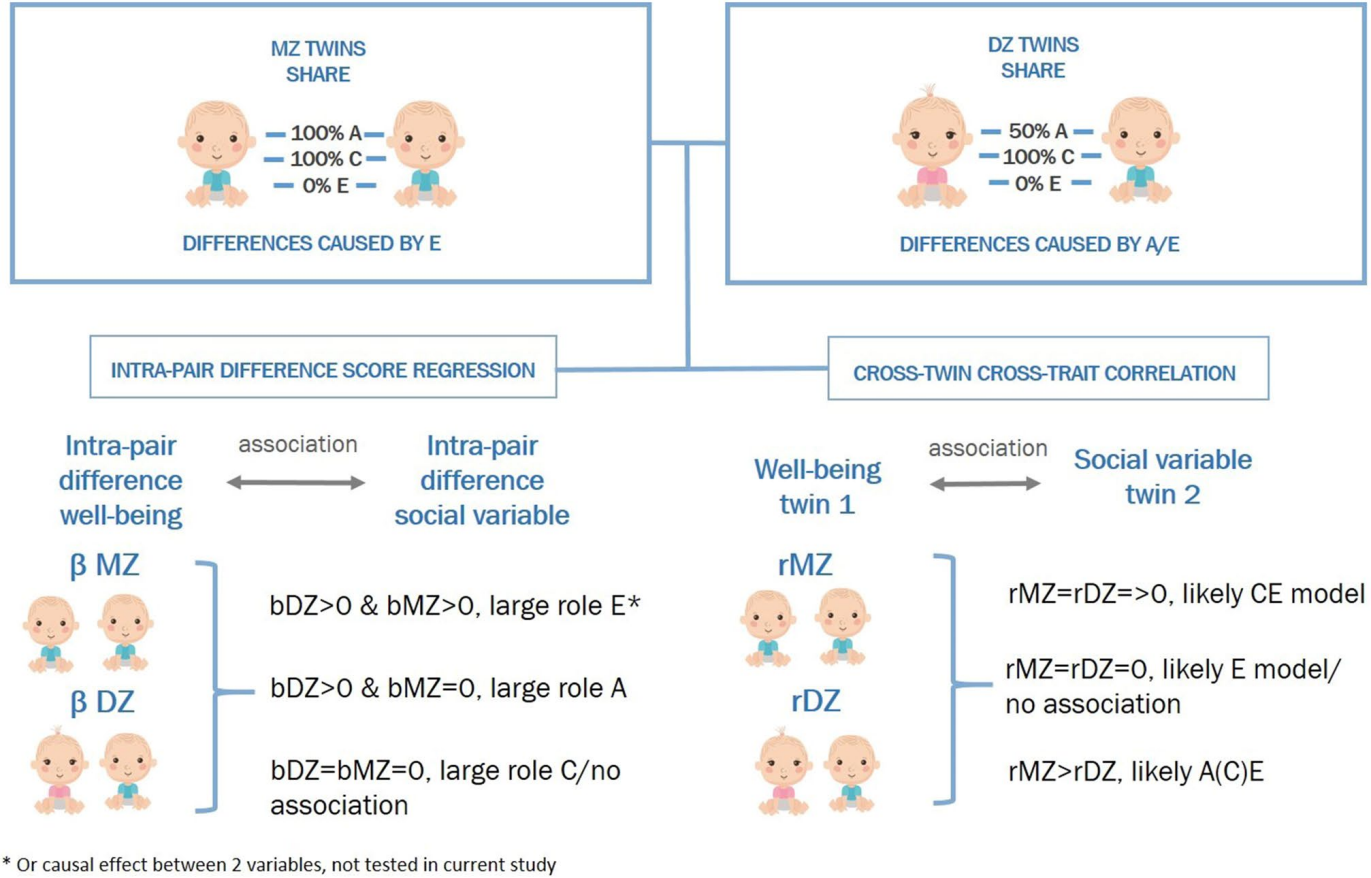
Overview of expectations based on DZ and MZ twin similarity and differences

All MZ CT–CT correlations were significantly different from zero. DZ correlations were either non-significant or smaller than MZ correlations for all traits except family conflict in males, suggesting that A has a substantial influence on the covariance between well-being and the social variables (see Fig. 2). For family conflict in males, MZ and DZ CT–CT correlations were of similar magnitude, suggesting a potential role for C on the covariance. The bivariate model fit comparisons can be found in eTable 4 in the Online Resources. For all traits, constraining the variance components to be equal across sex resulted in a significantly worse model fit. The full model variance decompositions can be found in eTable 5 in the Online Resources. In the full models, the C component for well-being and SWF in males is negative, likely due to genetic dominance [31].

**Fig. 3:**
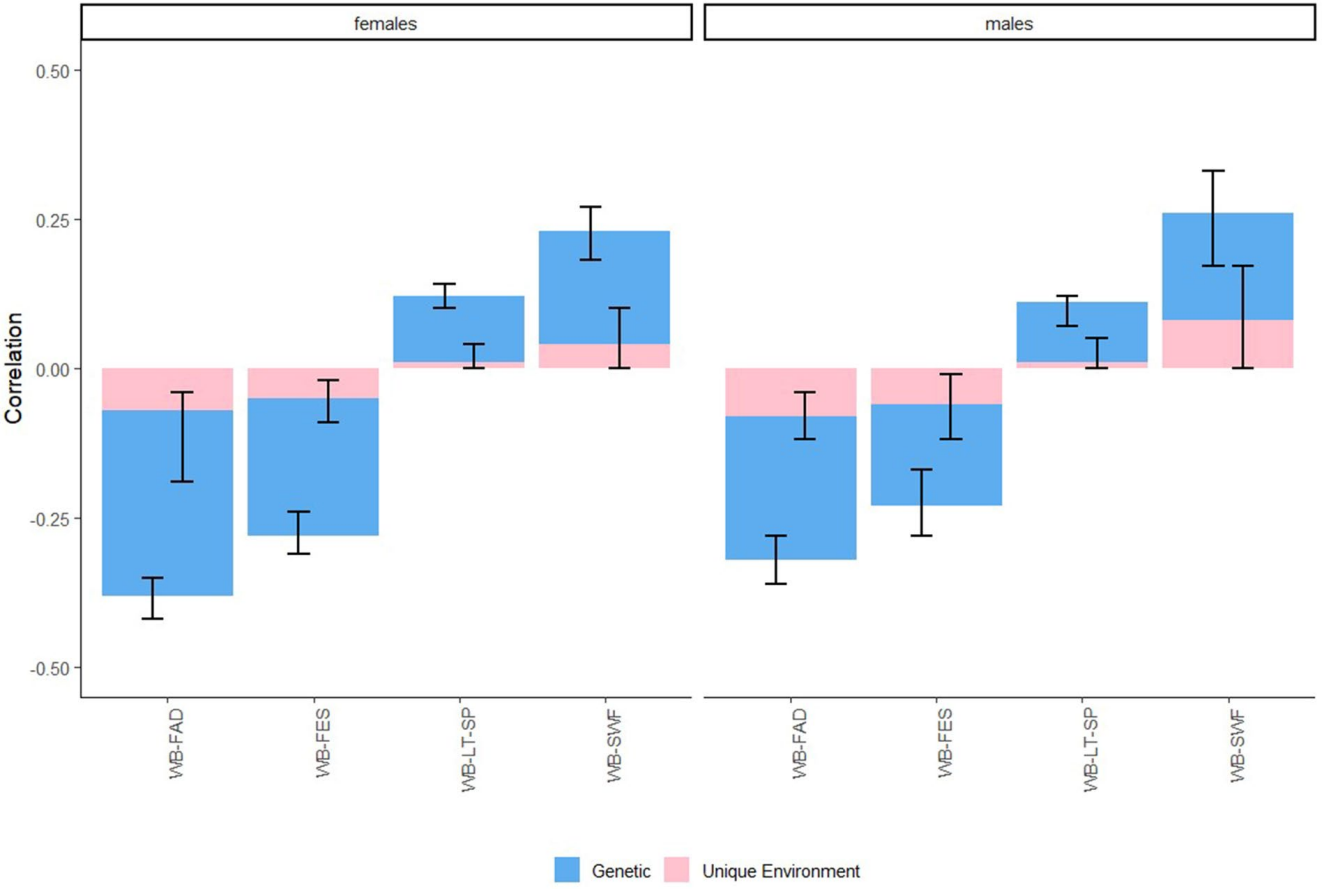
The contribution of genetic and environmental factors to correlations between well-being (WB) and family functioning (FAD), family conflict (FES), leisure time sport/scouting club (LT-SP), and satisfaction with friendships (SWF)

The final model (co)variance decomposition results for all traits can be found in Fig. 3 and Table 2. Across all bivariate models, C could be dropped for well-being and for the covariance between well-being and the social trait in question. Well-being heritability estimates (A) were in line with the previous studies [18], and we found slightly lower estimates for males (*A* = 35%, CI 29–41%) than females (*A* = 43%, CI 39–47%). In the bivariate model with well-being and FAD scores, all C components could be dropped for males, while the C component for FAD could not be dropped in females (C = 20%, CI = 8–32%). The heritability (A) of FAD was 47% (CI 41–52%) for males and 25% (CI 11–39%) for females. The covariance with well-being was mainly explained by genetic factors (males: 76%, CI = 63–89%, females: 73%, CI 49–95%). For FES scores, the best fitting model in males was an AE model (*A* = 55%, CI = 49–61%), while the best fitting model for females was an ACE model (*A* = 24% [CI 11–37%], *C* = 41% [CI = 29–52%]). The phenotypic correlation between well-being and FES was explained mostly by genetic factors (males: 73%, CI 49–95%, females: 81%, CI 69–93%).

**Table 2:** Standardized covariation decomposition of SWL with the different traits

	A		C		E	
	Social trait	WB	Social trait	WB	Social trait	WB
Males						
FAD	.47 [.41–.52]		–		.53 [.48–.59]	
WB	.76 [.63–.89]	.35 [.29–.41]	–	–	.24 [.11–.37]	.65 [.59–.71]
Females						
FAD	.25 [.11–.39]		.20 [.08–.32]		.55 [50–.60]	
WB	.82 [.74–.90]	.43 [.39–.47]	–	–	.18 [.11–.26]	.57 [.53–.61]
Males						
FES	.55 [.49–.61]		–		.45 [.39–.51]	
WB	.73 [.49–.95]	.35 [.29–.41]	–	–	.27 [.05–.51]	.65 [.59–.71]
Females						
FES	.24 [.11–.37]		.41 [.29–.52]		.35 [.31–.40]	
WB	.81 [.69–.93]	.43 [.39–.47]	–	–	.19 [.07–.31]	.57 [.53–.61]
Males						
SWF	.25 [.12–.38]		–		.75 [.62–.88]	
WB	.70 [.34–1.08]	.35 [.29–.41]	–	–	.30 [− .08 –.66]	.65 [.59–.71]
Females						
SWF	.34 [.24–.43]		–		.66 [.57–.76]	
WB	.82 [.58–.1.07]	.44 [.39–.48]	–	–	.18 [− .07–.42]	.56 [.52–.61]
Males						
LT–SP	.60 [.56–.64]		–		.40 [.36–.44]	
WB	.91 [.60–1.26]	.35 [.29–.41]	–	–	.09 [− .26–.40]	.65 [.59–.71]
Females						
LT–SP	.33 [.22–.44]		.33 [.23–.43]		.34 [.31–.37]	
WB	.89 [.69–1.08]	.43 [.39–.48]	–	–	.11 [–.08 –.31]	.57 [.52–.61]

*WB* well-being, *FAD* family functioning, *FES* family conflict, *SWF* satisfaction with friendships, *LT-SP* leisure time sport/scouting club, *A* additive genetic factors, *C* common environment, and *E* unique environment

For SWF, all C components could be dropped. The heritability (A) of SWF was estimated at 25% (CI 12–38%) in males and 35% (CI 29–41%) in females. Again, genetic factors explained the largest part of the covariation with well-being (males: 70%, CI 34–108%, females: 82%, CI 58–107%). Finally, for leisure time sport/scouting clubs, C could be dropped for males, but not females (*C* = 33%, CI 23–43%). The heritability (A) was estimated at 60% (CI 56–64%) in males, and 33% (CI 22–44%) in females. Genetic factors contributed to 91% of the covariance between well-being and leisure time sports/scouting clubs in males (CI 60–126%) and to 89% of the covariance in females (CI 69–108%). The estimates of our variance components were unbounded, which led to confidence intervals outside the usual range of 0–1 for these last two traits. This, together with the twin and CT–CT correlations, indicates a potential role for D.

eTable 6 (Online Resources) contains the genetic and environmental correlations between well-being and the other traits for males (*r*_Am_ and *r*_Em_, respectively) and females (*r*_Af_ and *r*_Ef_, respectively) separately. All genetic correlations were significant, with negative genetic correlations between well-being and FAD (*r*_Am_ = − 0.60, *r*_Af_ = − 0.94) and FES (*r*_Am_ = − 0.52, *r*_Af_ = − 0.71), and positive genetic correlations between well-being and SWF (*r*_Am_ = 0.54 *r*_Af_ = 0.54) and leisure time sports/scouting club (*r*_Am_ = 0.22 *r*_Af_ = 0.30). Unique environmental correlations were significant for FAD (*r*_Em_ = − 0.13, *r*_Ef_ = − 0.12) and FES (*r*_Em_ = − 0.12, *r*_Ef_ = − 0.12) only.

## Discussion

In the present study, we examined the relation between adolescent well-being and various social variables. We identified significant associations between well-being and family functioning, family conflict, leisure time indoor games, leisure time contact with friends, leisure time sports club/scouting, number of friends, and satisfaction with friendships. Well-being was not associated with leisure time crafts, leisure time making music/choir, leisure time spend on the computer, leisure time going out, leisure time watching TV, and importance of friendships.

Adolescent leisure time physical activity [33, 34], different aspects of adolescent friendships [35], and going out [36] have all previously been associated with well-being, just as familial and friendship variables [37–39]. These studies did not, however, examine potential genetic influences on these associations. Based on earlier research indicating that well-being and socio-environmental factors are subject to heritable influences [16, 18], we hypothesized that the observed associations might be partially explained by genetic factors. Intra-pair difference score analyses indicated genetic influences on the association between well-being and leisure time spend at sport/scouting clubs, and satisfaction with friendships. Intra-pair difference score associations for family functioning and family conflict suggested a role for both genetic and unique environmental influences. Moreover, these analyses reveal that genes do not seem to play a substantial role in the association of well-being with leisure time indoor games, leisure time contact with friends, and number of friends. Based on the difference score analyses, the relation between well-being and those three variables is most likely explained by common environmental influences. With respect to those friendship variables, a potential explanation is that it is siblings close in age spend time with the same peers. Additionally, parents might stimulate contact with peers through stimulating them to participate in outdoor activities, or alternatively limit time spent with peers based on how strict they are. With respect to leisure time indoor games, which includes chess, board games, and reading: the extent to which these things are present in a household is highly influenced by parents, which explains a large role for common environmental influences.

For traits where there was an indication that genetic factors played a role in the association with well-being (i.e., family functioning, family conflict, satisfaction with friendships, and leisure time spend at sport/scouting clubs), we performed bivariate genetic analyses. Common environmental influences did not contribute to associations with well-being, with genetic and unique environmental factors explaining the associations fully. For all traits, the largest part of the association was explained by genetic factors (between 73 and 91%). For females, a higher proportion of the association between well-being and the social traits was explained by genetic factors. For males, twin correlations indicated that D might contribute to variation in well-being and satisfaction with friendships. Additionally, CT–CT correlations for satisfaction with friendships and leisure time sport/scouting club also indicate a potential role for D. This is in line with the previous studies on well-being in adolescence, where a role for D was also indicated [28, 40].

While we did not directly test for causality in this study, we can draw some inferences based on the genetic and environmental correlations. If there is a causal relation between two traits, it is expected that genetic and environmental factors influencing one trait also influence the other trait (i.e., the genetic and environmental correlation should be significant [41]). If the genetic correlation is significant but the environmental correlation is not, this falsifies the hypothesis of a causal effect. In line with the difference score analyses, we found significant genetic correlations but non-significant unique environmental correlations between well-being and satisfaction with friendships and leisure time sports/scouting club, indicating that genetic factors play a dominant role in these associations. Additionally, we found significant genetic and unique environmental correlations between well-being and family conflict and family functioning, supporting a role for causality in these associations. Yet, it is important to mention that the significant unique environmental correlations with both FAD and FES were small (*r*_Em_ = − 0.13, *r*_Ef_ = − 0.12 and *r*_Em_ = − 0.12, *r*_Ef_ = − 0.12, respectively). While this does not falsify the claim that there might be a role for causality, this does indicate that a potential causal association will likely also be of small magnitude. Interestingly, we did not find a significant unique environmental correlation between well-being and satisfaction with friendships, suggesting that the association between those two traits is non-causal, at least in adolescence. While multiple studies identify an association between well-being and friendship quality/satisfaction [38, 42], these studies did not yet take into account the potential role of genetic factors. Based on what we find here, the most likely explanation for this association is that those who consider themselves to be satisfied with their lives are more likely to also consider themselves satisfied with their friendships due to them having a general (genetic) predisposition for positive ratings of life domains. This does not have to come as a surprise, since it has been shown that several well-being domains, such as satisfaction with life, satisfaction with friendship, and happiness are significantly associated both phenotypically as well as genetically [28, 43]. To check if our averaging the friendship variables over gender did not impact our conclusions, we performed supplementary GEE analyses where we examined same-sex and opposite-sex associations in males and females separately. Our results did not change when we examined these associations separately, even though the association between well-being and satisfaction with friendships was somewhat stronger for same-sex friendships than opposite-sex friendships (see Online Resource eTable 7).

These findings show that there is an important third factor in the association between well-being and several social variables in adolescence that is often unmeasured in psychological research: heritable influences. Phenotypic associations between well-being and different social variables are often found, but it appears that large parts of these associations are attributable to genetic factors. An interpretation of this genetic overlap is that the association between well-being and these variables is likely largely due to a genetic predisposition for appraising one’s life positively or negatively. For example, one might evaluate his or her well-being and friendship environment more positively in general because of their genetic predisposition for doing so. While this only pertains to the traits we now studied in more detail (i.e., family environment, friendship satisfaction, and leisure time sport/scouting club), an interesting question for future research would be to study if this genetic influence is also present for associations with other social variables (e.g., perceived social support in adolescence). Additionally, genetic and environmental correlations indicated that causality might be at play in the associations between well-being and family conflict and family functioning, with similar genetic and environmental factors influencing both traits, potentially through a causal chain.

An interesting follow-up for these findings is longitudinal studies, preferably using genetically sensitive designs. For example, if twin data are available, direction of causation models (if there are different modes of inheritance for the traits under study) [44] and genetic cross-lagged models [45] provide genetically sensitive methods for studying causality. In the absence of family data, one can still try to separate genetic from environmental effects if DNA data are available, for example by incorporating the effect of polygenic scores (scores that reflect individuals’ genetic predisposition for a trait based on results from genome-wide association studies) in mediation models [46] or Mendelian randomization models [47]. From a research perspective, it is important that investigations into adolescent mental health correlates take into consideration that these associations might reflect a shared genetic liability. In this study, we aimed to provide more information on these genetic influences, and confirmed that these cannot be ignored while studying these traits. This is also important from a clinical perspective, as the aim is to identify modifiable environmental factors in adolescence that improve well-being. What is important to keep in mind is that the mechanism behind (adolescent) well-being is very complex and multifaceted, with every relevant part only inducing a small, if any, change. Based on our results, the family environment seems a valuable part of the “well-being mechanism” that potentially has a small causal influence. This is interesting from an intervention perspective. However, as with any complex mechanism, the influence of a single aspect cannot be interpreted separate from all other effects. This means that its influence is different for different types of people, with strong causal effects being unlikely. Moreover, in this system, we cannot yet say anything about the potential direction of causality: while the family environment might influence well-being, this might also be the other way around or bidirectional. Moreover, the Netherlands is a country with relatively high levels of individualism according to Hofstede's individualism index [48], and it is important to interpret our findings within this a Western context, where relationships and group prosperity have a lower priority [49] than an Eastern context. Satisfaction with life is also known to be more suitable to measure well-being in the context of Western compared to Eastern cultures [50]. An interesting endeavor for future research would thus be to see how these associations vary across cultures and measures of well-being on both a phenotypic and genetic level.

In conclusion, we examined associations between well-being and a set of socio-environmental variables and find that genetic factors play a large role in several of these associations, confirming the importance of taking genetic differences into account. Additionally, we find a potential role for causality in the association between family conflict/functioning and well-being, with overlap in the genetic and environmental factors that influence these traits. From a clinical perspective, the family environment thus forms an interesting target for improving adolescent well-being.

## Supplementary Information

Below is the link to the electronic supplementary material.

Supplementary file1 (DOCX 79 KB)

## Data Availability

The Netherlands Twin Register cohort data may be accessed through the Netherlands Twin Register (ntr.fgb@vu.nl) upon approval of the data access committee.
