# Supplementary material for: Genetic factors explain a significant part of associations between adolescent well-being and the social environment
Source: Eur Child Adolesc Psychiatry. 2021 May 24;31(10):1611–22. doi: 10.1007/s00787-021-01798-3 (PMC9532338; doi:10.1007/s00787-021-01798-3)
Supplement: Supplementary file 1 — Supplementary file1 (DOCX 79 KB) [file 787_2021_1798_MOESM1_ESM.docx]

**Supplement to:**

**Genetic factors explain a significant part of associations between adolescent well-being and the social environment.**

M.P. van de Weijer^1,2^, MSc, dr. D. H. M. Pelt^1,2^, dr. C.E.M. van Beijsterveldt^1,2^, prof. dr. G. Willemsen^1.2^, prof. dr. M. Bartels^1,2^

^1^ Department of Biological Psychology, Vrije Universiteit Amsterdam, Amsterdam, The Netherlands

^2^ Amsterdam Public Health Research Institute, Amsterdam University Medical Center, Amsterdam, the Netherlands

**Content:**

**eFigure 1.** Illustration of the full covariance decomposition.

**eTable 1.** Complete twin pairs per zygosity group and variable.

**eTable 2.** Bivariate saturated model comparison results with well-being.

**eTable 3.** Correlations per zygosity group.

**eTable 4.** Bivariate genetic model fit comparisons (with well-being).

**eTable 5.** Standardized covariation decomposition of SWL with the different traits in the full ACE models.

**eTable 6.** Genetic and environmental correlations with well-being.

**eTable 7.** Phenotypic correlations between well-being and importance of/satisfaction with friendships separated for gender

**eFigure 1.** Illustration of the full covariance decomposition.

**
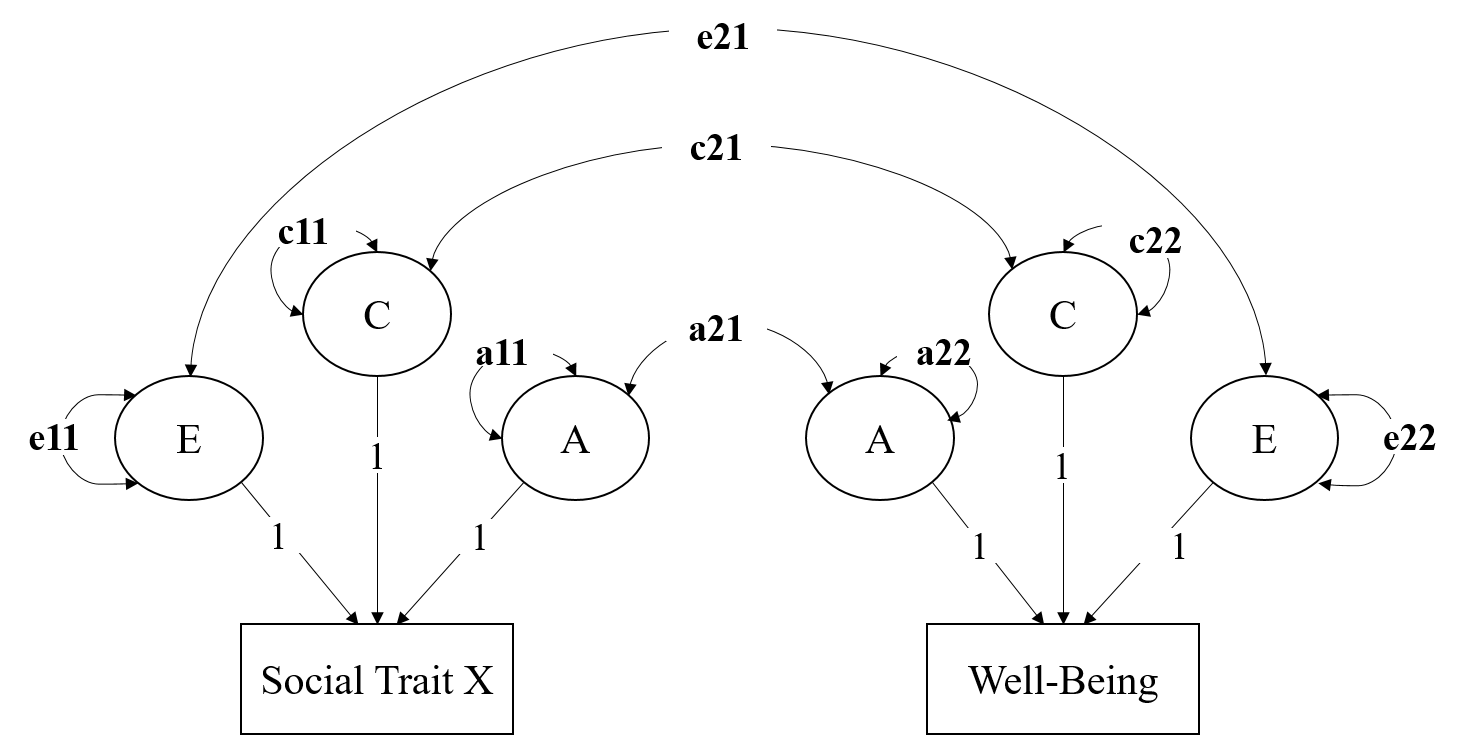
**

**eTable 1.** Complete twin pairs per zygosity group and variable.

|  | **Zygosity, 5 groups** | | | | | | **MZ** | | | **DZ** | | |
| --- | --- | --- | --- | --- | --- | --- | --- | --- | --- | --- | --- | --- |
|  | **N MZM** | **N DZM** | **N MZF** | **N DZF** | **N DOS** | **N total** | ***M* (*SD*) T1** | ***M* (*SD*) T2** | ***M* (*SD*) diff** | ***M* (*SD*) T1** | ***M* (*SD*) T2** | ***M* (*SD*) diff** |
| Well-being scores | 756 | 613 | 1118 | 835 | 1417 | 4739 | 26.67 (5.05) | 27.46 (5.24) | .21 (5.59) | 27.43 (4.99) | 27.44 (5.27) | -.01 (6.50) |
| Family functioning scores | 645 | 545 | 953 | 722 | 1229 | 4094 | 21.22 (5.28) | 21.39 (5.39) | -.17 (5.60) | 21.65 (5.37) | 21.52 (5.44) | .14 (6.42) |
| Family conflict scores | 421 | 357 | 669 | 511 | 839 | 2797 | 14.13 (2.34) | 14.13 (2.40) | .003 (2.13) | 14.09 (2.40) | 14.06 (2.41) | .02 (2.48) |
| Leisure time – indoor games | 710 | 584 | 1060 | 790 | 1333 | 4477 | 5.12 (2.24) | 5.17 (2.28) | -.05 (2.18) | 5.13 (2.27) | 5.06 (2.23) | .07 (2.72) |
| Leisure time - contact with friends | 676 | 566 | 1040 | 793 | 1333 | 4408 | 10.55 (3.54) | 10.57 (3.52) | -.02 (3.16) | 10.82 (3.39) | 10.84 (3.52) | -.02 (3.95) |
| Leisure time - crafts | 709 | 588 | 1066 | 798 | 1358 | 4519 | 4.47 (2.68) | 4.66 (2.77) | -.18 (2.80) | 4.73 (2.76) | 4.60 (2.78) | .14 (3.52) |
| Leisure time - making music/choir | 720 | 594 | 1080 | 805 | 1373 | 4572 | 2.16 (1.86) | 2.20 (1.92) | -.04 (1.67) | 2.14 (1.84) | 2.13 (1.84) | .01 (2.18) |
| Leisure time - computer | 293 | 229 | 386 | 269 | 508 | 1685 | 9.47 (2.47) | 9.49 (2.42) | -.02 (2.14) | 9.57 (2.42) | 9.64 (2.57) | -.07 (3.11) |
| Leisure time - going out | 721 | 596 | 1088 | 810 | 1378 | 4593 | 2.43 (1.30) | 2.44 (1.31) | -.01 (.99) | 2.44 (1.32) | 2.43 (1.32) | .02 (1.23) |
| Leisure time - sport/scouting club | 727 | 592 | 1079 | 809 | 1375 | 4584 | 3.86 (1.77) | 3.88 (1.75) | -.02 (1.50) | 3.96 (1.71) | 3.97 (1.75) | -.003 (1.91) |
| Leisure time - TV | 303 | 233 | 398 | 286 | 522 | 1742 | 6.54 (.96) | 6.49 (1.04) | .05 (.82) | 6.52 (.88) | 6.49 (.91) | .03 (1.04) |
| Number of friends | 438 | 367 | 705 | 537 | 867 | 2914 | 5.17 (2.96) | 5.35 (3.16) | -.17 (2.98) | 5.38 (3.01) | 5.43 (3.02) | -.06 (3.61) |
| Importance of friendships | 82 | 63 | 127 | 85 | 145 | 502 | 3.17 (.60) | 3.22 (.70) | -.05 (.73) | 3.16 (.68) | 3.22 (.72) | -.06 (.94) |
| Satisfaction with friendships | 319 | 272 | 544 | 395 | 664 | 2194 | 3.28 (.78) | 3.32 (.75) | -.04 (.97) | 3.31 (.71) | 3.28 (.79) | .03 (1.05) |
| *Note.* *N*= N twin pairs. MZM= monozygotic males, DZM= dizyotic males, MZF= monozygotic females, DZF = dizygotic females, DOS= dizygotic opposite sex twins, *M*= mean, *SD*= standard deviation, T1 = twin 1, T2= twin 2, diff= difference score. | | | | | | | | | | | | |
|  |  |  |  |  |  |  |  |  |  |  |  |  |

**eTable 2.** Bivariate saturated model comparison results with well-being.

| **Model** | **Comparison** | **-2LL** | **df** | **AIC** | **Δ-2LL** | **Δ df** | **p** |
| --- | --- | --- | --- | --- | --- | --- | --- |
| *Family functioning (FAD) scores* |  |  |  |  |  |  |  |
| 1. Full model |  | 125785.14 | 20704 | 84377.14 |  |  |  |
| 2. eq. means twin order | 1. Full model | 125801.75 | 20712 | 84377.75 | 16.60 | 8 | 0.035 |
| 3. 2+ eq. var twin order | 1. Full model | 125817.98 | 20720 | 84377.98 | 32.83 | 16 | 0.008 |
| 4. 3+ eq. across zygosity | 1. Full model | 123828.71 | 20728 | 84372.71 | 43.56 | 24 | 0.009 |
| 5. 4 + eq. across SS/OS | 1. Full model | 125844.47 | 20736 | 84372.41 | 59.33 | 32 | 0.002 |
| 6. 5 + eq. across sex | 1. Full model | 125949.48 | 20740 | 84469.48 | 164.33 | 36 | 2.59x10^-18^ |
| *Family conflict (FES) scores* |  |  |  |  |  |  |  |
| 1. Full model |  | 9817791 | 17957 | 62263.91 |  |  |  |
| 2. eq. means twin order | 1. Full model | 98188.94 | 17965 | 62258.94 | 11.03 | 8 | 0.200 |
| 3. 2+ eq. var twin order | 1. Full model | 98205.88 | 17973 | 62259.88 | 27.97 | 16 | 0.032 |
| 4. 3+ eq. across zygosity | 1. Full model | 98215.64 | 17871 | 62253.64 | 37.72 | 24 | 0.037 |
| 5. 4 + eq. across SS/OS | 1. Full model | 98234.31 | 17879 | 62256.31 | 56.39 | 32 | 0.005 |
| 6. 5 + eq. across sex | 1. Full model | 98337.74 | 17993 | 62351.74 | 159.83 | 36 | 1.55x10^-17^ |
| *Satisfaction with friendships* |  |  |  |  |  |  |  |
| 1. Full model |  | 79058.63 | 16473 | 46112.63 |  |  |  |
| 2. eq. means, var & thresholds twin order | 1. Full model | 79091.14 | 16489 | 46113.14 | 32.51 | 16 | 0.009 |
| 3. 2 + eq. across zygosity | 1. Full model | 79097.73 | 16497 | 46103.73 | 39.10 | 24 | 0.027 |
| 4. 3 + eq. across SS/OS | 1. Full model | 79112.48 | 16505 | 46102.48 | 53.85 | 32 | 0.009 |
| 5. 4 + eq. across sex | 1. Full model | 79254.82 | 16509 | 46236.82 | 196.19 | 36 | 6.10x10^-24^ |
| *Leisure time sport/scouting club* |  |  |  |  |  |  |  |
| 1. Full model |  | 108571.48 | 21785 | 65001.48 |  |  |  |
| 2. eq. means twin order | 1. Full model | 108581.84 | 21793 | 64995.84 | 10.36 | 8 | 0.241 |
| 3. 2+ eq. var twin order | 1. Full model | 108597.67 | 21801 | 64995.67 | 26.18 | 16 | 0.051 |
| 4. 3+ eq. across zygosity | 1. Full model | 108610.79 | 21809 | 64882.79 | 39.30 | 24 | 0.025 |
| 5. 4 + eq. across SS/OS | 1. Full model | 108623.60 | 21817 | 64989.60 | 52.12 | 32 | 0.014 |
| 6. 5 + eq. across sex | 1. Full model | 108749.17 | 21821 | 65107.17 | 177.69 | 36 | 1.21x10^-20^ |
|  |  |  |  |  |  |  |  |

**eTable 3.** Correlations per zygosity group.

|  | **WB** | **FAD** | **FES** | **SWF** | **LT-SP** |
| --- | --- | --- | --- | --- | --- |
|  |  | *within-twin within-trait correlation* | | | |
| **MZM** | .37 (.30 to .42) | .46 (.39 to .51) | .55 (.48 to .61) | .30 (.15 to .43) | .60 (.55 to .63) |
| **DZM** | .16 (.08 to .24) | .26 (.18 to .33) | .40 (.30 to .48) | .01 (0 to .16) | .37 (.30 to 43) |
| **MZF** | .43 (.39 to .48) | .45 (.40 to .49) | .64 (.60 to .68) | .35 (.25 to .45) | .66 (.63 to .69) |
| **DZF** | .28 (.21 to.34) | .37 (.31 to .43) | .55 (.49 to .60) | .20 (.07 to .33) | .50 (.45 to .54) |
| **DZO** | .19 (.14 to .23) | .27 (.22 to .32) | .45 (.40 to .50) | .16 (.06 to .26) | .35 (.30 to .39) |
|  |  | *cross-twin cross-trait correlation with well-being* | | | |
| **MZM** |  | -.23 (-.31 to -.15) | -.18 (-.28 to -.07) | .21 (.05 to .35) | .08 (.01 to .16) |
| **DZM** |  | -.17 (-.26 to -.08) | -.20 (-.30 to -.08) | .04 (0 to.19) | -.03 (-.11 to 0) |
| **MZF** |  | -.28 (-.33 to -.22) | -.21 (-.28 to -.14) | .18 (.06 to .29) | .13 (.07 to .18) |
| **DZF** |  | -.21 (-.28 to -.14) | -.10 (-.18 to -.01) | .08 (0 to .20) | .08 (.01 to .15) |
| **DZO** |  | -.16 (-.21 to -.11) | -.12 (-.19 to -.05) | .09 (0 to .18) | .06 (.01 to .11) |

**eTable 4.** Bivariate genetic model fit comparisons (with well-being).

| **Model** | **Comparison** | **-2LL** | **df** | **AIC** | **Δ-2LL** | | **Δ df** | **p** |
| --- | --- | --- | --- | --- | --- | --- | --- | --- |
| *Family functioning (FAD) scores* | |  |  |  |  |  | |  |
| 1. ACE, sex differences |  | 38428.44 | 14181 | 10066.44 |  |  | |  |
| 2. ACE, no sex differences | 1. ACE, sex differences | 38471.33 | 14190 | 10091.33 | 42.89 | 9 | | 2.26x10^-6^ |
| 3. 1 + drop c22 | 1. ACE, sex differences | 38431.00 | 14183 | 10065.00 | 2.56 | 2 | | 0.278 |
| 4. 3 + drop c21 | 1. ACE, sex differences | 38439.11 | 14185 | 10069.11 | 10.66 | 4 | | 0.031 |
| 5. 4 + drop c11 | 1. ACE, sex differences | 38449.59 | 14187 | 10075.59 | 21.15 | 6 | | 0.002 |
| **6. 4 + drop c11 males** | **1. ACE, sex differences** | **38439.30** | **14186** | **10067.30** | **10.86** | **5** | | **0.054** |
| 7. 4 +drop c11 females | 1. ACE, sex differences | 38449.40 | 14186 | 10077.40 | 20.96 | 5 | | 0.001 |
| *Family conflict(FES) scores* | |  |  |  |  |  | |  |
| 1. ACE, sex differences |  | 33255.28 | 12298 | 8659.28 |  |  | |  |
| 2. ACE, no sex differences | 1. ACE, sex differences | 33313.26 | 12307 | 8699.26 | 57.97 | 9 | | 3.29x10^-9^ |
| 3. 1 + drop c22 | 1. ACE, sex differences | 33258.10 | 12300 | 8658.10 | 2.82 | 2 | | 0.244 |
| 4. 3 + drop c21 | 1. ACE, sex differences | 33265.53 | 12302 | 8661.53 | 10.25 | 4 | | 0.036 |
| 5. 4 + drop c11 | 1. ACE, sex differences | 33307.33 | 12304 | 8699.33 | 52.05 | 6 | | 1.83x10^-9^ |
| **6. 4 + drop c11 males only** | **1. ACE, sex differences** | **33269.32** | **12303** | **8663.32** | **14.04** | **5** | | **0.015** |
| 7. 4 + drop c11 females only | 1. ACE, sex differences | 33303.54 | 12303 | 8697.54 | 48.26 | 5 | | 3.15x10^-9^ |
| *Satisfaction with friendship scores* | |  |  |  |  |  | |  |
| 1. ACE, sex differences |  | 28651.56 | 11305 | 6041.56 |  |  | |  |
| 2. ACE, no sex differences | 1. ACE, sex differences | 28688.52 | 11314 | 6060.52 | 36.96 | 9 | | 2.68x10^-5^ |
| 3. 1 + drop c22 | 1. ACE, sex differences | 28654.16 | 11307 | 6040.16 | 2.60 | 2 | | 0.273 |
| 4. 3 + drop c21 | 1. ACE, sex differences | 28654.39 | 11309 | 6036.39 | 2.83 | 4 | | 0.587 |
| **5. 4 + drop c11** | **1. ACE, sex differences** | **28657.42** | **11311** | **6035.42** | **5.86** | **6** | | **0.439** |
| *Leisure time sport/scouting club* | |  |  |  |  |  | |  |
| 1. ACE, sex differences |  | 40421.51 | 14926 | 10569.51 |  |  | |  |
| 2. ACE, no sex differences | 1. ACE, sex differences | 40470.08 | 14935 | 10600.08 | 48.57 | 9 | | 2.00x10^-7^ |
| 3. 1 + drop c22 | 1. ACE, sex differences | 40424.33 | 14928 | 10568.33 | 2.82 | 2 | | 0.244 |
| 4. 3 + drop c21 | 1. ACE, sex differences | 40425.70 | 14930 | 10565.70 | 4.19 | 4 | | 0.380 |
| 5. 4 + drop c11 | 1. ACE, sex differences | 40463.14 | 14932 | 10599.14 | 41.63 | 6 | | 2.17x10^-7^ |
| **6. 4 + drop c11 males only** | **1. ACE, sex differences** | **40428.77** | **14931** | **10566.77** | **7.26** | **5** | | **0.202** |
| 7. 4 + drop c11 females only | 1. ACE, sex differences | 40460.07 | 14931 | 10598.07 | 38.56 | 5 | | 2.91x10^-7^ |
| *Note*. The best fitting model is indicated in **bold,** c22= c component well-being c21= C covariance between well-being and the social trait, c11= c component social trait | | | | | | | | |
|  |  |  |  |  |  |  |  |  |

**eTable 5.** Standardized covariation decomposition of SWL with the different traits in the full ACE models.

|  |  | A | | C | | E | |
| --- | --- | --- | --- | --- | --- | --- | --- |
|  |  | **Social trait** | **WB** | **Social trait** | **WB** | **Social trait** | **WB** |
| males | **FAD** | .40 [.21-.59] |  | .06 [-.10-.22] |  | .54 [.48-.60] |  |
|  | **WB** | .57 [.11-1.04] | .39 [.19-.59] | .17 [-.23-.56] | -.04 [-.21-.13] | .26 [.12-.41] | .65 [.59-.71] |
| females | **FAD** | .16 [.01-.31] |  | .29 [.16-.41] |  | .55 [.50-.60] |  |
|  | **WB** | .36 [.07-.67] | .32 [.17-.47] | .42 [.15-.67] | .11 [-.04-.24] | .22 [.13-.31] | .57 [.53-.62] |
| males | **FES** | .32 [.11-.53] |  | .22 [.03-.40] |  | .46 [.40-.53] |  |
|  | **WB** | .22 [-.58-1] | .40 [.20-.60] | .46 [.40-.53] | -.04 [-.22-.13] | .32 [.08-.58] | .64 [.58-.71] |
| females | **FES** | .18 [.05-.31] |  | .47 [.35-.57] |  | .35 [.31-.40] |  |
|  | **WB** | .26 [-.16 - .68] | .31 [.16-.47] | .51 [.14-.86] | .11 [-.03-.25] | .23 [.11-.36] | .58 [.53-.62] |
| males | **SWF** | .58 [.15-.99] |  | -.28 [-.62 -.07] |  | .70 [.57-.84] |  |
|  | **WB** | .49 [-.73-1.68] | .39 [.20-.60] | .17 [-.81-1.17] | -.04 [-.21-13] | .34 [-.06-.73] | .64 [.58-.71] |
| females | **SWF** | .31 [-.02 - .64] |  | .03 [-.25 -.29] |  | .67 [.57-.77] |  |
|  | **WB** | .62 [-.20 - 1.48] | .32 [.16-.47] | .18 [-.53 -.86] | .11 [-.03-.24] | .20 [-.08-.45] | .57 [.53-.62] |
| males | **LT-SP** | .46 [.31-.62] |  | .14 [-.01 -.27] |  | .40 [.36-.45] |  |
|  | **WB** | .52 [-.72 -1.74] | .41 [.21-.61] | .36 [-.71-1.45] | -.05 [-.22-.12] | .12 [-.24-.45] | .63 [.58-.70] |
| females | **LT-SP** | .32 [.21-.43] |  | .34 [.24-.44] |  | .34 [.31-.37] |  |
|  | **WB** | .47 [-.26 - 1.23] | .31 [.16-.47] | .39 [-.29-1.03] | .11 [-.03-.24] | .14 [-.05-.34] | .57 [.53-.62] |
| *Note.* WB=well-being, FAD= family funtioning, FES= family conflict, SWF= satisfaction with friendships, LT-SP = leisure time sport/scouting club | | | | | | | |
|  |  |  |  |  |  |  |  |

**eTable 6.** Genetic and environmental correlations with well-being.

|  | **FAD** | **FES** | **SWF** | **LT-SP** |
| --- | --- | --- | --- | --- |
|  | *Genetic correlations* | | | |
| r_Am_ | -.55 [-.67 to -.44] | -.36 [-.50 to -.22] | .58 [.30 to .78] | .21 [.11 to .31] |
| r_Af_ | -.68 [-.76 to -.61] | -.72 [-1 to -53] | .50 [.34 to .67] | .28 [.17 to .41] |
|  | *Environmental correlations* | | | |
| r_Em_ | -.13 [-.20 to -.06] | -.14 [-.24 to -.04] | .11 [0 to .23] | .02 [-.05 to .10] |
| r_Ef_ | -.12 [-.18 to -.07] | -.13 [-.20 to -.05] | .09 [-.01 to .19] | .03 [-.03 to .09] |
| ***Note*.** FAD=family functioning, FES=family conflict, SWF=satisfaction with friendships, LT-SP= leisure time sport/scouting club. | | | | |
|  |  |  |  |  |

| **eTable 7**. Phenotypic correlations between well-being and importance of/satisfaction with friendships separated for gender | | | |
| --- | --- | --- | --- |
|  | **GEE (whole sample)** | | |
|  | β (SE) | p | N |
| Importance of female friendships in females | 0.05 (.04) | 0.125 | 1367 |
| Importance of male friendships in females | 0.02 (.03) | 0.486 | 1208 |
| Importance of female friendships in males | 0.03 (.03) | 0.365 | 832 |
| Importance of male friendships in males | 0.03 (.03) | 0.398 | 956 |
| *Importance of friendships average* | *0.06 (.03)* | *0.062* | *1322* |
| Satisfaction with female friendships in females | 0.18 (.02) | 3.99x10^-24^ | 4565 |
| Satisfaction with male friendships in females | 0.11 (.02) | 3.67x10^-11^ | 4032 |
| Satisfaction with female friendships in males | 0.14 (.02) | 3.73x10^-11^ | 2682 |
| Satisfaction with male friendships in males | 0.18 (.02) | 2.60x10^-17^ | 3169 |
| *Satisfaction with friendships average* | *0.16 (.01)* | *9.33x10^-27^* | *6519* |
